# Isolation, Screening, and Degradation Characteristics of a Quinclorac-Degrading Bacterium, Strain D, and Its Potential for Bioremediation of Rice Fields Polluted by Quinclorac

**DOI:** 10.1128/Spectrum.00398-21

**Published:** 2021-09-29

**Authors:** Siqi Huang, Jiuyue Pan, Mancuo Tuwang, Hongyan Li, Chenyi Ma, Mingxue Chen, Xiaoyan Lin

**Affiliations:** a Rice Product Quality Inspection and Supervision Center, China National Rice Research Institute, Hangzhou, China; University of Minnesota

**Keywords:** quinclorac, *Cellulosimicrobium cellulans* strain D, degradation characteristics, field effect evaluation, bioremediation, pesticide

## Abstract

Quinclorac (QNC) is a persistent, highly selective, hormonal herbicide of low toxicity. QNC accumulates in soil and affects the growth and development of crops planted subsequent to its application. In this study, we isolated and screened a QNC-degrading bacterial strain, strain D, from rice paddy soil. Morphological analysis, physiological and biochemical tests, and 16S rRNA gene sequencing led us to identify strain D as a Cellulosimicrobium cellulans strain. We investigated the characteristics of strain D in relation to QNC degradation. Under optimal culture conditions, the QNC degradation rate was 45.9% after 21 days of culture. QNC degradation by strain D in the field was modeled and quantified by a pot experiment. The results show that strain D promotes rice growth and degrades QNC. This research has identified a new bacterial species that degrades QNC, providing a foundation for further research into QNC remediation.

**IMPORTANCE** QNC-degrading bacteria have been isolated from different environments, but there are no reports of Cellulosimicrobium cellulans strains that degrade QNC. In this study, a previously unidentified bacterial strain that degrades QNC, strain D, was screened from paddy soil. The characteristics of strain D that relate to QNC degradation were investigated in detail. The results showed that strain D effectively degraded QNC. Two degradation products of QNC formed by strain D that have not been reported previously, i.e., 3-pyridylacetic acid (*m/z* 138.0548) and 3-ethylpyridine (*m/z* 108.0805), were identified using high-performance liquid chromatography–quadrupole time of flight mass spectrometry. Strain D has the capacity to degrade QNC in a QNC-polluted paddy.

## INTRODUCTION

Quinclorac (QNC) is a highly selective persistent hormonal herbicide of low toxicity. It affects plant metabolism and growth by altering plant hormones. It is principally used to control barnyard grass in rice fields (1). QNC has been widely used because it is an effective herbicide that can be applied over a long period of time. In 2016, QNC was one of the top 10 herbicides used in rice paddies in China; annual sales reached $41.21 million (2). Long-term use of QNC results in it accumulating in soil and affecting the growth and development of crops subsequently planted in the QNC-contaminated soil (3, 4). Tobacco-rice crop rotation is widely used in Hunan Province, Jiangxi Province, Guangdong Province, and other regions of China. Herbicide in which QNC exceeded 25% of the manufacturer-recommended dose that was applied in the rice season was found to harm subsequent tobacco crops by increasing the numbers of deformed leaves and reducing the yield (5–7). Residual phytotoxicity of QNC seriously affects Solanaceae crops, Umbelliferae crops, and Chenopodiaceae crops. Crops such as potatoes, peppers, carrots, celery, spinach, and beets were found to be sensitive to the herbicide, although they were not planted until at least 2 years after the application of QNC (8–10). Residual QNC in the environment has been found to adversely affect the growth and development of animals, to cause abnormal development or reproductive abnormalities in animals, and to affect the diversity and abundance of many natural organisms (11). The wide application of QNC in rice production has drawn increasing attention to the harmful effects of QNC on animals and plants. In view of its long persistence and ecological toxicity, it is increasingly necessary to find a practical and effective method of degrading residual QNC to remediate the environmental pollution that QNC causes.

The principal methods of QNC degradation that are currently being explored are chemical degradation and biodegradation. Chemical methods mainly include photodegradation and electrodynamic soil cleansing. Titanium dioxide has been used as a photocatalyst for QNC degradation. TiO_2_ P25 nanoparticles were used to photocatalyze the degradation of QNC in ultrapure water irradiated by an 1,100-W xenon lamp. Degradation was complete after 40 min; however, complete degradation took 130 min when paddy water was used (12, 13). The photodegradation products were complex, and QNC was not fully mineralized. Light absorption of TiO_2_ P25 is limited to the UV region (320 to 400 nm), and UV light accounts for only 5% of the solar spectrum. Thus, most investigations into photocatalytic degradation of pollutants use irradiation from high-power xenon lamps or UV lamps. These techniques require a lot of electrical energy, which makes large-scale experiments unfeasible. Electrokinetic soil cleansing (14) seemed at its inception to be an effective technology for remediation of pesticide-polluted soil. However, during the electrochemical process, soil pH is changed, which leads to the degradation of agricultural soil. In addition, pollutants that were removed from the soil were subsequently dissolved in the soil moisture during the electrokinetic process, and additional treatment was required to eliminate pollutants from the moisture. This was a time-consuming and complex process. Biodegradation seems to be a more effective approach. Microorganisms have diverse metabolic pathways and extensive substrates and create few by-products, which suggests that microorganisms may have a range of uses in pesticide degradation.

QNC-degrading bacteria have been isolated from various environments. *Bordetella* sp. strain HN36 degrades QNC and also quinoline, phthalic acid, phenol, and catechol (15). A pot experiment showed that *Alcaligenes* sp. strain J3 remediated compounds that had phytotoxic effects on tobacco (16). A bioremediation experiment using *Pantoea* sp. strain QC06 showed that it significantly remediated compounds that had phytotoxic effects on tobacco and thus might be capable of degrading QNC residues in tobacco fields (17). *Arthrobacter* sp. strain MC-10 was found to degrade QNC residues in contaminated soil within 7 days (18). Li et al. showed that 3-chloro-7-hydroxyquinine-8-carboxylic acid and 7-chloro-3-hydroxyquinine-8-carboxylic acid were degradation products of Mycobacterium sp. strain F4 and were nonphytotoxic to tobacco (19). Lang et al. screened *Streptomyces* sp. strain AH-B through circulating fluidized bed culturing and enrichment and found that the main degradation products were 3-chloro-7-methoxy-8-quinoline carboxylic acid, 3-chloro-7-methyl-8-quinoline carboxylic acid, 3-chloro-7-oxyethyl-8-quinoline-carboxylic acid, and 3,7-dichloro-6-methyl-8-quinoline carboxylic acid (20). Zhou et al. found that Stenotrophomonas maltophilia strain J03 in minimal salt medium (MSM) degraded 33.5% of QNC after 21 days and the strain was effective in alleviating the harmful effects of QNC on tobacco plant growth (21). The QNC-degrading bacteria screened in most studies were used to remediate compounds that had phytotoxic effects on tobacco. There have been few studies of the remediation of residual QNC in rice paddy soil. The purpose of this study was to screen QNC-degrading bacteria that had adapted to the paddy environment, to determine whether they could be widely used to remediate QNC-contaminated paddies.

To date, there have been no reports of Cellulosimicrobium cellulans strains that degrade QNC. Current research shows that C. cellulans strains decompose cellulose, alkane powder, gelatin, xylan, paraffin, and fixed nitrogen (22); for example, Liang et al. isolated and screened out the C. cellulans strain DGNK-JJ1, which dissolves potassium. Provision of effective strains for the production of potassium-dissolving bacterial fertilizers has contributed to the development of ecological agriculture (23). Ferrer showed that C. cellulans strains can be used as biocontrols for plant diseases (24). These studies have shown that these kinds of microorganisms can be used in fertilizers, for plant protection, and to extract protective agents and they are thus likely to develop into a fruitful avenue of biological research. In this study, we found that C. cellulans strains had a previously unknown capacity for QNC degradation. In this study, we first isolated and screened a strain, strain D, that can use QNC as the only carbon source from paddy soil. Strain D was identified as a C. cellulans strain. We experimentally determined the degradation effect, degradation characteristics, and degradation products of strain D. The effectiveness of the strain was quantified by a pot experiment that modeled rice paddies.

## RESULTS AND DISCUSSION

### Identification of strain D.

In our experiment, we screened and isolated a bacterial strain, strain D, that degraded QNC. The colony was round, light yellow, and moist and had smooth surfaces and neat edges. The cells were rod-shaped, with swollen cysts or spores and thicker capsules, in a chain-like arrangement, and were Gram positive. The physiological and biochemical properties are shown in Table 1. The glucose, d-ribose, and contact enzyme test results were positive, and the raffinose test results were negative. This result is consistent with the description of C. cellulans in the eighth edition of *Bergey’s Manual of Determinative Bacteriology* (25).

**TABLE 1:** Physiological and biochemical characteristics of strain D

Test	Result^*a*^
β-Galactosidase	+
Arginine	−
Lysine	−
Ornithine	+
Citric acid	−
Hydrogen sulfide	−
Urease	−
Lactose	−
Indole	+
Voges-Proskauer	+
Gelatin	−
Glucose	+
Mannitol	−
Inositol	−
Sorbitol	−
l-Rhamnose	−
Sucrose	+
Melibiose	−
Amygdalin	+
Arabinose	+
Oxidase	−
d-ribose	+
Raffinose	−
Catalase	+

a +, positive; −, negative.

**FIG 1:**
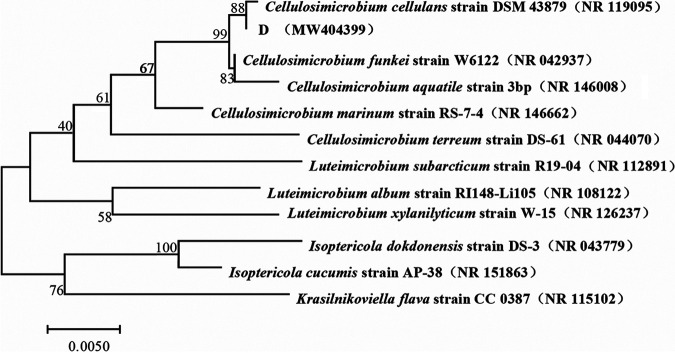
Phylogenetic tree of the 16S rRNA gene of strain D.

The 16S rRNA sequence of strain D was uploaded to NCBI for nucleotide sequence comparison, and a phylogenetic tree was constructed. The 16S rRNA gene phylogenetic tree of strain D is shown in Fig. 1. Strain D (1,384 bp) (GenBank accession no. MW404399) and C. cellulans strain DSM 43879 (GenBank accession no. NR_119095) are clustered in the same branch of the 16S rRNA phylogenetic tree, with homology of 99.93%. Therefore, strain D was identified as a C. cellulans strain.

### Characteristics of QNC degradation by strain D. (i) Effect of initial pH on QNC degradation.

**FIG 2:**
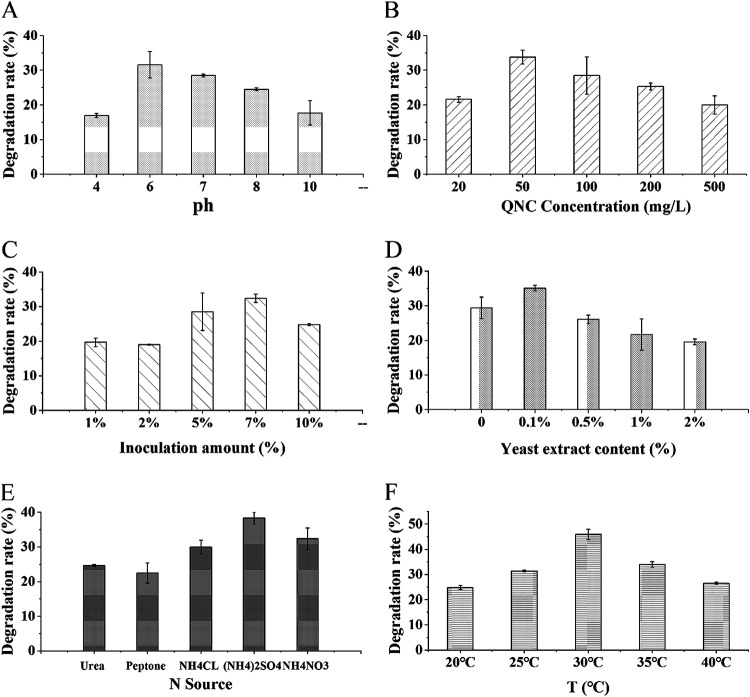
Factors affecting QNC degradation. (A) Effect of initial pH on QNC degradation by strain D. (B) Effect of initial concentration of QNC on QNC degradation by strain D. (C) Effect of inoculation amount on QNC degradation by strain D. (D) Effect of yeast extract content on QNC degradation by strain D. (E) Effect of external nitrogen source on QNC degradation by strain D. (F) Effect of temperature on QNC degradation by strain D. Error bars indicate standard deviations (*n *= 3).

Strain D had little effect in degrading QNC in strongly acidic (pH < 4) or strongly alkaline (pH > 10) environments, as shown in Fig. 2A. Strain D degraded QNC most effectively in a weakly acidic environment; when the culture solution had a pH of 6, the degradation rate was 31.6%. QNC contains easily hydrolyzable carboxyl groups and has a pH of 4.35, which is weakly acidic (26). QNC dissociates easily under alkaline conditions, and acidic conditions inhibit its dissociation. A suitable pH value for rice growth is 6 to 7; therefore, QNC does not dissociate easily in paddy soil. The strains selected in this study degraded QNC most effectively when the pH was 6 to 7, which makes them very suitable for degrading QNC in rice fields.

### (ii) Effect of initial QNC concentration on QNC degradation.

Degradation of QNC by strain D in liquid MSM with initial concentrations of QNC of 20, 50, 100, 200, and 500 mg · liter^−1^ is shown in Fig. 2B. It can be seen that, when QNC was employed as the only carbon source, QNC could be degraded to different degrees by strain D. The greatest degradation occurred when the concentration of QNC was 50 mg · liter^−1^; strain D degraded 33.8% of the QNC. QNC was the only carbon source for strain D and thus greatly influenced degradation by strain D. The percent degraded increased as the QNC concentration increased within the range of 20 to 50 mg · liter^−1^. When the concentration became too great, the percent degraded decreased. We explain this as being due to the high physiological toxicity of QNC, which inhibits the reproduction and metabolism of strain D. Lv (27) showed that, when QNC concentrations exceed a certain limit, microorganisms experience oxidative stress. The optimal initial mass concentration of QNC for satisfactory degradation is reported to be in the range of 5 to 1,000 mg · liter^−1^. S. maltophilia strain J03 (21) degrades 33.5% of QNC at an initial mass concentration of 5 mg · liter^−1^ over 21 days. Burkholderia cepacia strain WZ1 (27) degrades 90% of QNC over 5 days. However, bacteria that degrade QNC when the concentration of QNC is low or optimal are not suitable for application to QNC-contaminated farmland, where the manufacturer-recommended rate of QNC application is 375 g · hm^−2^ · a^−1^. The optimal QNC concentration of strain D that we identified was close to the QNC concentration in seriously polluted paddy soil, which implies that strain D is suitable for application in heavily polluted paddies.

### (iii) Effect of inoculum quantity on QNC degradation.

The effects of inoculum quantity on the degradation of QNC are shown in Fig. 2C. Microorganism growth was affected by the quantity of the inoculum, which in turn affected the growth and reproduction of bacterial strains. When the inoculum quantity was small, its effect on the bacterial culture was delayed; as the inoculum quantity was increased, the rate of QNC degradation increased. Strain D had the greatest effect on degradation when the inoculum quantity was 7%, and at that level it continuously degraded QNC at a steady rate; 32.4% of QNC was degraded after culturing for 21 days. When the inoculum quantity exceeded 7%, the QNC degradation rate decreased, which we attribute to there being a dynamic equilibrium between bacterial population growth and decreasing nutrient availability.

### (iv) Effect of nitrogen source on QNC degradation.

The type and content of nutrients affect the degradation ability of microorganisms. By optimizing the N source, degradation by strain D could be increased. The effect of different N sources on the degradation of QNC is shown in Fig. 2D. It can be seen that degradation of QNC changed according to the different nitrogen sources in the culture medium. The degradation of QNC promoted by different N sources for strain D was ordered as follows: (NH_4_)_2_SO_4_ > NH_4_NO_3_ > NH_4_Cl > urea > peptone. Thus, (NH_4_)_2_SO_4_ was selected as the optimal N source. Strain D showed the greatest degradation of QNC (38.3% in 21 days) when (NH_4_)_2_SO_4_ was used as the N source. When all other parameters were unchanged and the nitrogen source was (NH_4_)_2_SO_4_, the degradation rate for strain D could be increased by 5.9% to 15.8%. In addition, (NH_4_)_2_SO_4_ is an excellent nitrogen fertilizer that is suitable for general soil and crops. It promotes vigorous growth of branches and leaves, improves fruit quality and yield, and increases crop resistance to damage, and it can be used as base fertilizer, top dressing, or seed fertilizer; it is widely used in rice production.

### (v) Effect of yeast extract on QNC degradation.

Yeast extract is rich in protein, amino acids, peptides, nucleotides, B vitamins, and trace elements. It can be used to supplement N and C sources, and it contains vitamins and micronutrients that promote the growth of microorganisms (28). The addition of yeast extract could promote the growth and reproduction of microorganisms and thus increase QNC degradation. The effect of yeast extract on the degradation of QNC by strain D is shown in Fig. 2E. When the yeast extract content was 0.1%, degradation by strain D was at the maximum rate of 35.1%. The degradation effect at 21 days was worst when the quantity of yeast extract was 2%. We surmise that the relatively large quantity of yeast extract changed the degradation target of strain D.

### (vi) Effect of temperature on QNC degradation.

Temperature importantly affects the growth and survival of microorganisms. The effect of temperature on QNC degradation by strain D was mainly to alter the growth, reproduction, and metabolism of the bacteria. The optimal temperature for bacteria that degrade QNC has been found to be in the range of 25°C to 35°C. The degradation of QNC by strain D at different temperatures, when all other culture variables were unchanged, is shown in Fig. 2F. Fig. 2F shows that, as the culture temperature was increased, QNC degradation by strain D increased. Degradation was greatest when the temperature reached 30°C; 45.9% of the QNC was degraded. Degradation decreased as the temperature was increased above 30°C. Strain D had a degradation rate in the range of 31.4% to 45.9% when the temperature was in the range of 25°C to 35°C. This result indicates that strain D can adapt to the ground temperature during rice planting and growth.

### (vii) QNC degradation under optimal culture conditions.

**FIG 3:**
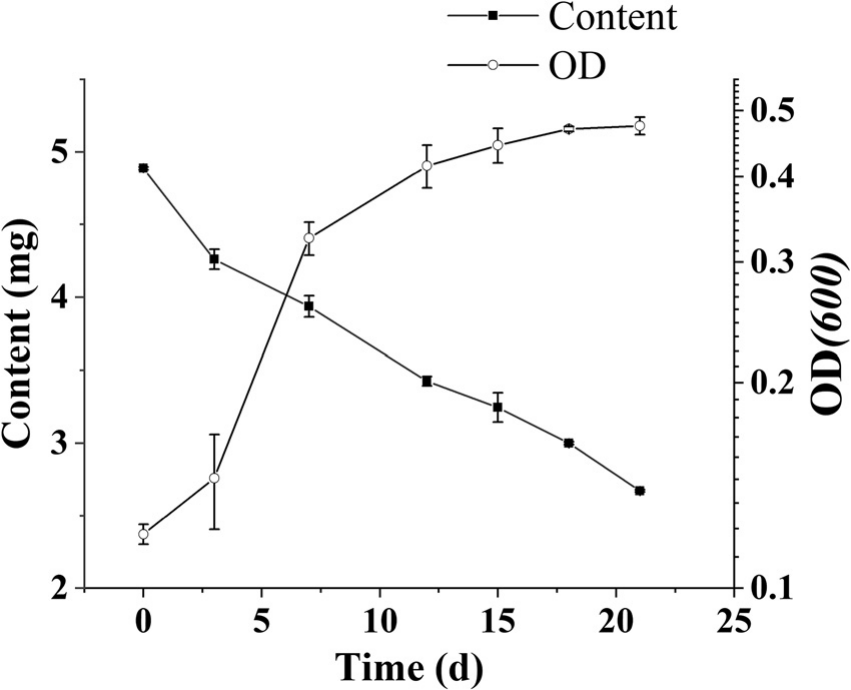
Effects of optimal conditions on the QNC degradation by strain D and the semilogarithmic growth curve of strain D. Data are mean ± standard error from three replicates.

Optimal culture conditions were as follows: initial pH, 6; initial QNC concentration, 50 mg · liter^−1^; inoculation quantity, 7%; yeast extract content, 0.1%; nitrogen source, (NH_4_)_2_SO_4_; culture temperature, 30°C; culture period, 21 days. The degradation of QNC by strain D and the strain D semilogarithmic growth curve when conditions were optimal are shown in Fig. 3. Strain D had degraded 45.9% of the QNC after being cultured for 21 days. Strain D entered the log phase of growth after being cultured for 3 days and entered the stable phase after 18 days. Under natural conditions, QNC is degraded only by light and microorganisms. QNC-degrading bacteria screened in existing studies were mostly used to remediate compounds that were phytotoxic to tobacco; there have been few studies on the remediation of residual QNC in paddy soil (17, 29). The purpose of this study was to screen QNC-degrading bacteria that were adapted to the paddy in order that the strain could be widely used in QNC-contaminated paddies. The degradation of QNC by strain D bacteria is influenced by many factors, including temperature, pH, initial substrate mass concentration, inoculum quantity, and the capacity of the strain to degrade QNC (30). We conducted a single-factor experiment to optimize the environmental conditions for degradation by strain D, and we found that the optimal conditions for strain D were similar to those found in a paddy environment and that strain D was thus adapted to the paddy environment.

### Analysis of the degradation products of QNC.

**FIG 4:**
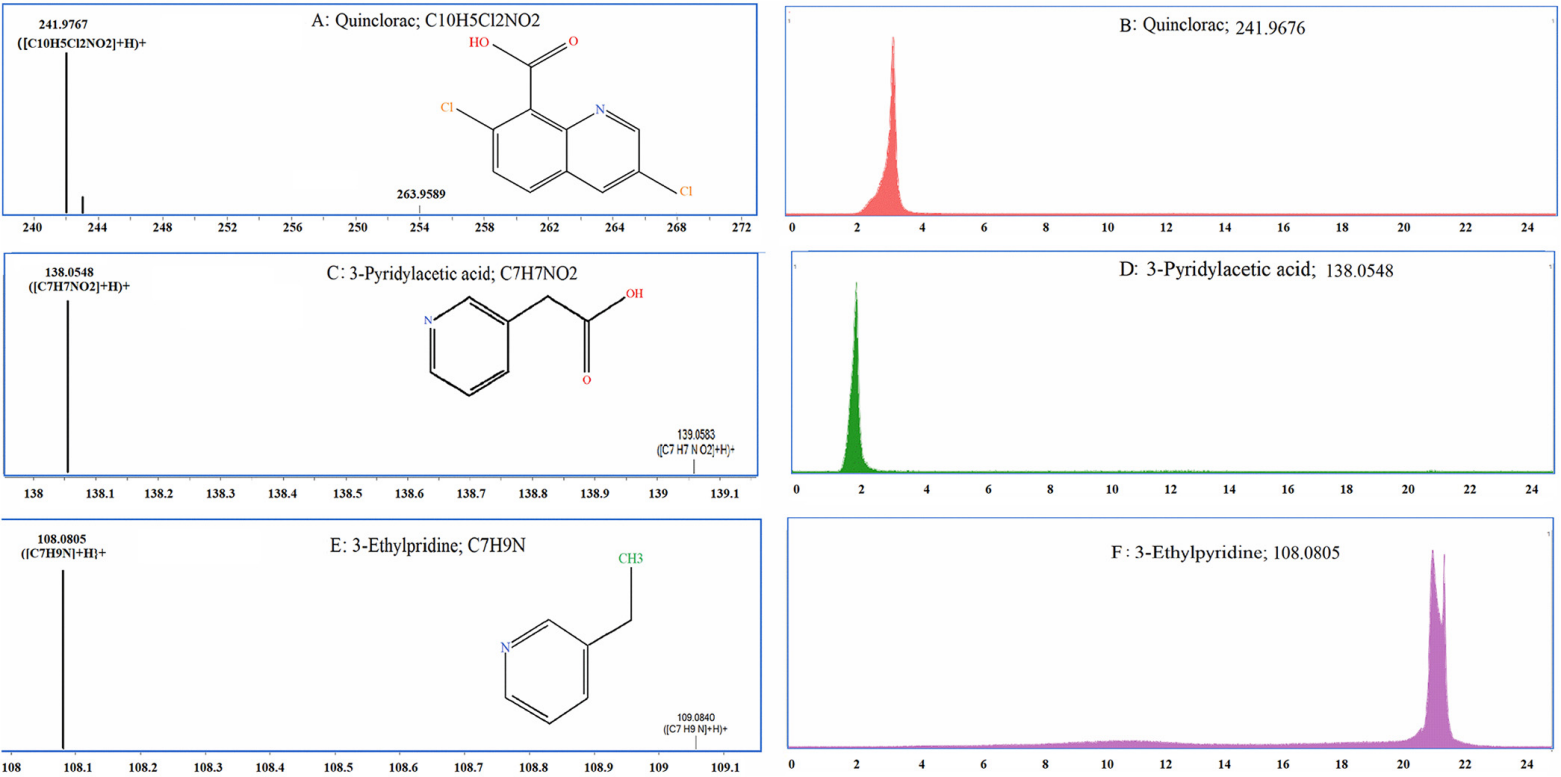
Mass spectra and chromatograms of QNC and QNC degradation products. (A) Mass spectrum of QNC. (B) Mass chromatogram of QNC. (C) Mass spectrum of 3-pyridylacetic acid. (D) Mass chromatogram of 3-pyridylacetic acid. (E) Mass spectrum of 3-ethylpyridine. (F) Mass chromatogram of 3-ethylpyridine.

The QuEChERS (quick, easy, cheap, effective, rugged, safe) method was used to extract QNC and its degradation products from the MSM at different stages of culturing. QNC and its degradation products were detected by high-performance liquid chromatography–quadrupole time of flight mass spectrometry (HPLC–Q-TOF MS). Two possible QNC metabolites were detected. The mass spectrum and chromatogram of the detected QNC and the two degradation products are shown in Fig. 4. The main degradation products identified by mass spectrometry were 3-pyridylacetic acid (*m/z* 138.0548) and 3-ethylpyridine (*m/z* 108.0805). The degradation products were not quantified due to a lack of standards. These degradation products have not been reported in previous studies of QNC degradation, and the degradation pathways and enzymes involved are still under analysis. We suspect that these compounds are probably new metabolites of QNC that was degraded by strain D. Further tests are needed to verify our hypothesis.

### Effects of strain D on QNC degradation in a simulated rice paddy.

**FIG 5:**
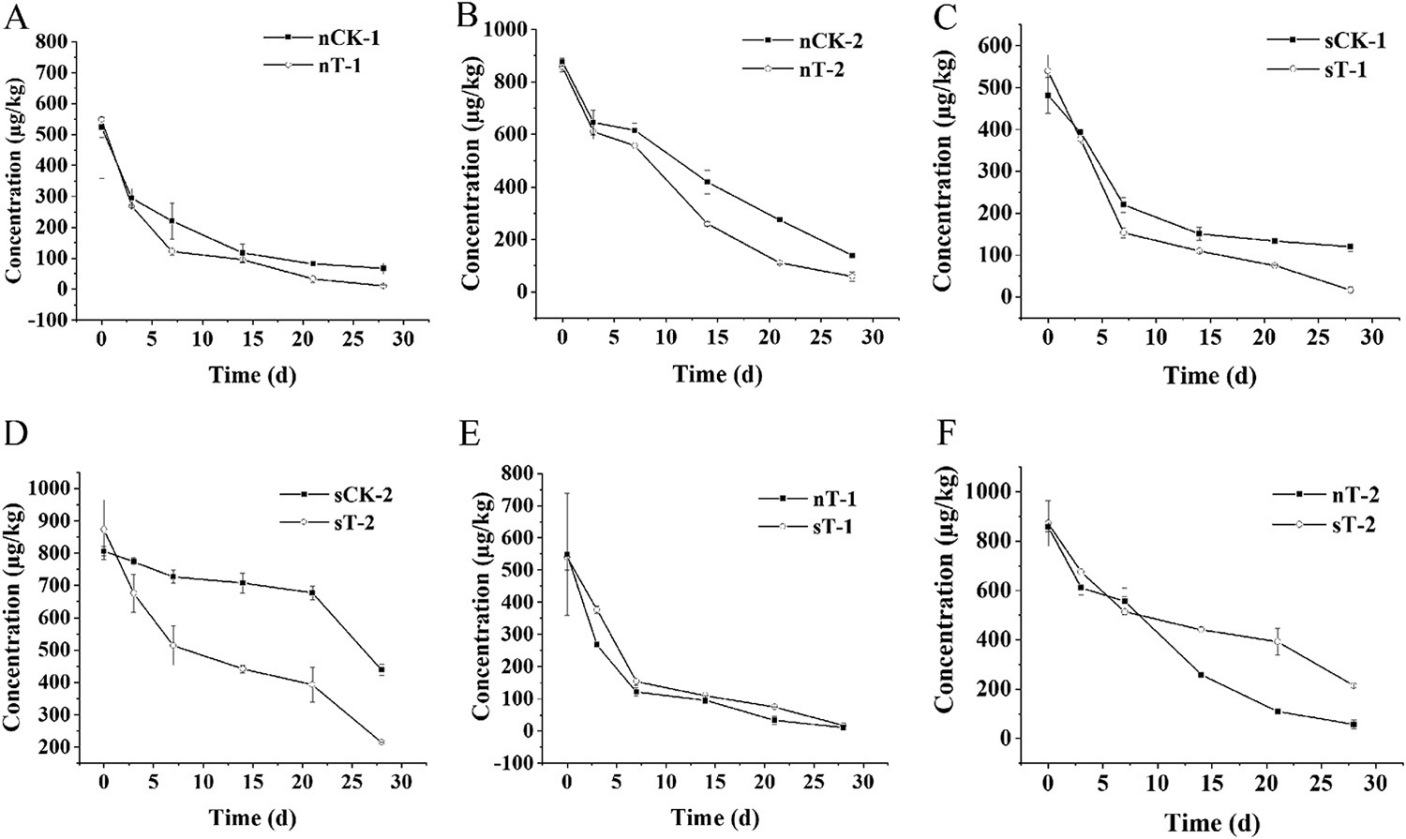
Degradation of QNC in soil. (A) Degradation for the recommended dose in normal soil. (B) Degradation for 2 times the recommended dose in normal soil. (C) Degradation for the recommended dose in sterilized soil. (D) Degradation for 2 times the recommended dose in sterilized soil. (E) Degradation for the experimental group with the recommended dose in sterilized soil and normal soil. (F) Degradation for the experimental group with 2 times the recommended dose in sterilized soil and normal soil. Data are mean ± standard error from three replicates.

The manufacturer-recommended dose (375 g · hm^−2^ · a^−1^) and 2 times the recommended dose (750 g · hm^−2^ · a^−1^) were applied in soil remediation pot experiments that simulated the rice paddy. In these experiments, some soil was sterilized and QNC-degrading strain D bacteria were added so that we could investigate field bioremediation of QNC. Degradation of QNC in the soil in each treatment group over 28 days is shown in Fig. 5. Experiments using normal soil (Fig. 5A and B) showed that strain D accelerated the degradation of QNC. Degradation increased when the QNC concentration was 750 g · hm^−2^ · a^−1^. Experiments using sterilized soil (Fig. 5C and D) showed that degradation of QNC in the control group (without degradation bacteria) was significantly less than that in sterilized soil with degradation bacteria. Strain D degraded 75.45% of QNC within 21 days with treatment with QNC at 750 g · hm^−2^ · a^−1^. It is clear that strain D is very suitable for remediation of a seriously QNC-polluted rice field. Comparison of the degradation of QNC in normal soil and in sterilized soil (Fig. 5E and F) clearly shows that QNC was degraded more rapidly in normal soil. There are two reasons for this. First, in the sterilized soil only strain D bacteria were active in degradation, whereas in normal soil there are exogenous bacteria that are also active in QNC degradation. If there is synergy between strain D and exogenous bacteria, QNC degradation will increase. Second, sterilization of the soil may alter its physical and chemical properties and thus reduce the activity of degradation bacteria. Strain D increased QNC degradation whether or not the soil was sterilized. This indicates that strain D is adapted to the paddy environment and will thus be effective in degrading QNC and so remediating QNC-contaminated rice fields.

### Effects of strain D on rice plants in a simulated rice paddy.

**FIG 6:**
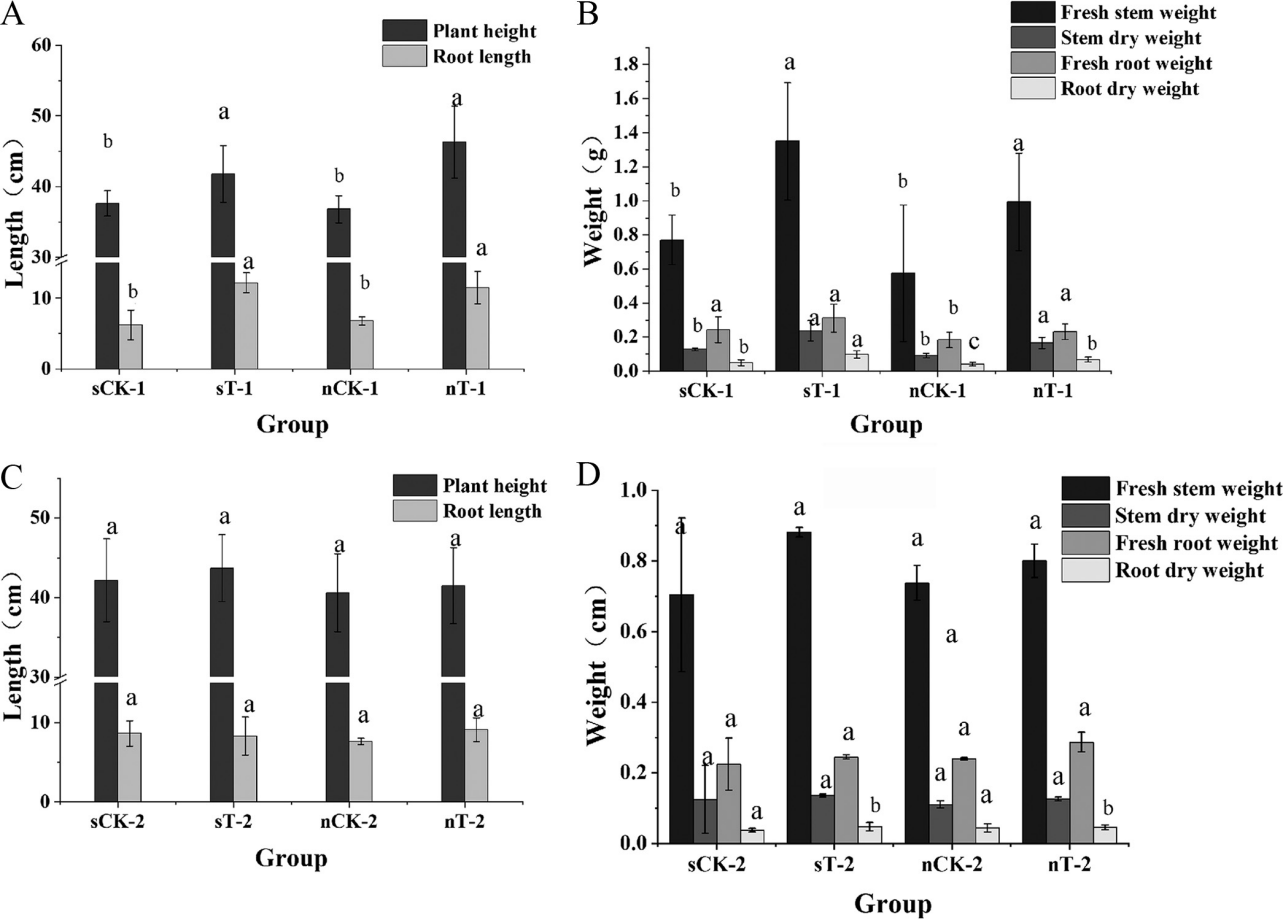
Growth of rice plants in simulated rice fields at 7 days. Different letters indicate significant differences at the *P* = 0.05 level. (A) Plant height and root length for the experimental group and the control group at the recommended dose (375 g · hm^−2^ · a^−1^). (B) Stem fresh weight, stem dry weight, root fresh weight, and root dry weight for the experimental group and the control group at the recommended dose (375 g · hm^−2^ · a^−1^). (C) Plant height and root length for the experimental group and the control group at 2 times the recommended dose (750 g · hm^−2^ · a^−1^). (D) Stem fresh weight, stem dry weight, root fresh weight, and root dry weight for the experimental group and the control group at 2 times the recommended dose (750 g · hm^−2^ · a^−1^). Data are mean ± standard error from three replicates.

**FIG 7:**
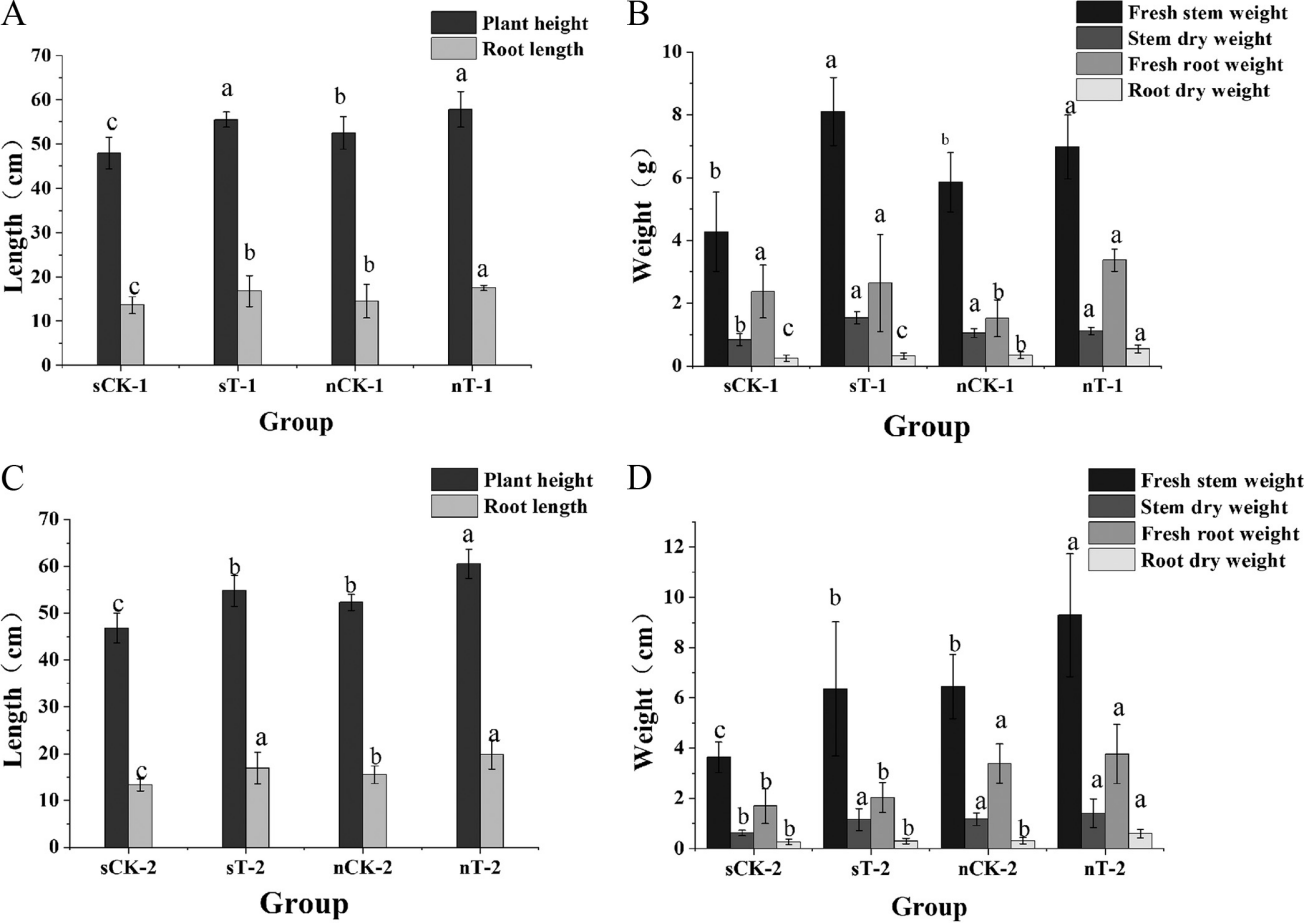
Growth of rice plants in simulated rice fields at 21 days. Different letters indicate significant differences at the *P* = 0.05 level. (A) Plant height and root length for the experimental group and the control group at the recommended dose (375 g · hm^−2^ · a^−1^). (B) Stem fresh weight, stem dry weight, root fresh weight, and root dry weight for the experimental group and the control group at the recommended dose (375 g · hm^−2^ · a^−1^). (C) Plant height and root height for the experimental group and the control group at 2 times the recommended dose (750 g · hm^−2^ · a^−1^). (D) Stem fresh weight, stem dry weight, root fresh weight, and root dry weight for the experimental group and the control group at 2 times the recommended dose (750 g · hm^−2^ · a^−1^). Data are mean ± standard error from three replicates.

Sensitive dicotyledons may be significantly affected by high concentrations of QNC and show this under conditions such as chlorosis, necrosis, and stunted growth (31). Rice has a relatively high tolerance for QNC, but excessive QNC use presents a higher risk of abnormal development in rice seedlings. We investigated the effects of strain D on rice plant physiology due to QNC stress using pot experiments to simulate rice paddies. In the experiments, the application of strain D had clear effects on the plants. Seven days after spraying, when the QNC concentration in the pot was at the manufacturer-recommended dose (375 g · hm^−2^ · a^−1^) (Fig. 6A and B), rice plant height in the experimental groups was 20.5% higher than that in the control group. The fresh stem weight for the experimental groups using sterilized soil was 64.3% greater than that for the sterilized soil control group. There were significant differences in dry root weight between the control group in the sterilized soil groups and the normal soil experimental groups when the QNC concentration in the pot was 2 times the manufacturer-recommended dose (750 g · hm^−2^ · a^−1^) (Fig. 6C and D). Although there were clear differences in the roots, no differences were observed in other parts of the plants. The reason for this may be that rice root exudates interacted with soil microorganisms in the rhizosphere to promote root growth (32). When the QNC concentration in the pot was 2 times the manufacturer-recommended dose (750 g · hm^−2^ · a^−1^) (Fig. 7C and D), the rice plant height, root length, and stem fresh weight for the sterilized soil and normal soil experimental groups were significantly different from those for the control group after 21 days. However, this was not the case on day 7 of the experiments. The reason for this may be that the higher concentration of QNC had a greater impact on plant physiology and the QNC-degrading bacteria needed more time to adapt to the environment before they became active. The root dry weight for the normal experimental group was 41.4% greater than that for the sterilized soil experimental group. This result was consistent with the previously observed phenomenon of more rapid degradation in the normal soil experimental group. The pot experiments show that QNC-degrading strain D significantly bioremediated QNC-polluted soil and strain D will thus promote the degradation of residual QNC and reduce its phytotoxicity to rice in a QNC-polluted paddy.

Since it was discovered that QNC is naturally degraded principally by microorganisms, many kinds of QNC-degrading bacteria have been screened. However, researchers have mainly investigated degradation characteristics, and there have been no studies of the bioremediation of QNC-contaminated rice fields by QNC-degrading bacteria; therefore, there is no groundwork for the future practical application of QNC-degrading bacteria. In this study, we used highly effective QNC-degrading bacteria and simulated rice fields to investigate the bioremediation of QNC-contaminated rice fields. The results of our experimental work show that QNC-degrading strain D bacteria will adapt to the rice field environment and synergistically degrade QNC in conjunction with naturally occurring rice field soil bacteria. The remediation reduces the adverse effects of QNC on rice plant physiology and growth and indicates that strain D has the potential for widespread application in remediating rice paddies.

## MATERIALS AND METHODS

### Materials.

The following materials were used in the study: QNC standard (98%; Shanghai Yuanye Biological Company), QNC wettable powder (50%; Jiangsu Kuaida Agrochemical Co., Ltd.), liquid LB medium (10 g tryptone, 5 g yeast extract, 10 g NaCl [pH 7.0], 1,000 ml distilled water), solid LB medium (10 g tryptone, 5 g yeast extract, 10 g NaCl, 20 g agar [pH 7.0], 1,000 ml distilled water), and liquid MSM (0.2 g MgSO_4_·7H_2_O, 0.2 g CaCl_2_, 1.0 g KH_2_PO_4_, 1.0 g Na_2_HPO_4_, 0.05 g FeCl_3_, 1.0 g NH_4_NO_3_, 1,000 ml distilled water); rice paddy soil and rice seeds were all acquired from China Rice Research Institute.

### Screening of QNC-degrading strains.

A sample of 5 g paddy soil was added to 100 ml liquid MSM with 100 mg · liter^−1^ QNC and incubated at 30°C and 180 rpm for 7 days. A 5-ml quantity of the medium was dispensed to a new liquid MSM sample with 200 mg · liter^−1^ QNC to be cultured. After 7 days, another 5 ml of the original MSM was dispensed. This process was repeated until the concentration of QNC in the receiving MSM reached 500 mg · liter^−1^. The final culture medium was gradient diluted and inoculated into solid LB medium, which was cultured at 30°C for 48 h. A colony was selected and streaked on solid LB medium to obtain single colonies. Different single colonies were subsequently selected and cultured in liquid MSM with 100 mg · liter^−1^ QNC for 21 days. QNC was extracted using QuEChERS liquid chromatography–tandem mass spectrometry, and the residual concentration of QNC was determined (33) to select the dominant candidate strain.

### Identification of QNC-degrading strains.

Solid LB medium was inoculated with the selected strain and cultured at 30°C for 48 h for observation of the morphological characteristics of the colony. The Gram positivity or Gram negativity of the strain was determined using Gram stain, and bacterial morphology was observed under a scanning electron microscope. Physiological and biochemical kits (Qingdao Haibo Biological Co., Ltd.) were used for physiological and biochemical identification. *Bergey’s Manual of Determinative Bacteriology* (25) was referred to for identification. After the strain D bacteria had been activated, a BigDye Terminator v3.1 cycle sequencing kit (Applied Biosystems Inc., USA) was used to extract the entire DNA of strain D. Agarose gel electrophoresis with a 1% gel was used to determine DNA extraction efficiency. Two universal primers (27F [5′-AGAGTTTGATCCTGGCTCAG-3′] and 1492R [5′-TACCTTGTTACGACTT-3′]) were used for the reaction. The amplification reaction components were 1 μl DNA, 8 μl BigDye, 1 μl primer (3.2 pmol · liter^−1^), and 10 μl sterile deionized water. The reaction protocol was as follows: predenaturation at 96°C for 1 min; 25 cycles of denaturation at 96°C for 10 s, annealing at 50°C for 5 s, and extension at 60°C for 4 min; and storage at 4°C. Amplification products were submitted to Shanghai Shangya Biotechnology Co., Ltd., for sequencing. The sequencing results were uploaded to the NCBI database, and the homology comparison was performed by BLAST in GenBank (http://www.ncbi.nlm.nih.gov). MEGA7.0 software was used for phylogenetic analysis, and the phylogenetic tree was constructed using the neighbor-joining method.

### Characteristics of QNC degradation by strain D. (i) Preparation of bacterial suspension.

Strain D was inoculated into 100 ml liquid LB medium, incubated at 30°C and 180 rpm for 48 h, and centrifuged at 8,000 rpm for 5 min, and the upper layer of LB medium was decanted. The precipitated strain was washed twice in sterile physiological saline solution, and the pellet was resuspended in liquid MSM (optical density at 600 nm [OD_600_] of 1.0) for later use.

### (ii) Effects of different culture conditions on QNC degradation.

We investigated the effects on QNC degradation of pH, initial QNC concentration, inoculum amount, yeast extract content, N source, and temperature as follows. The pH of the culture solution was adjusted to 4, 6, 7, 8, or 10, and the solution was cultured at 30°C. The liquid MSM was constituted with QNC concentrations of 20, 50, 100, 200, or 500 mg · liter^−1^, and the solution was cultured at 30°C. The initial inoculation amount of the culture solution was set to 1%, 2%, 5%, 7%, or 10%, and the solution was cultured at 30°C. Quantities of 0, 0.1%, 0.5%, 1%, or 2% yeast extract were used to investigate the effect of yeast extract on QNC degradation. Urea, peptone, (NH_4_)_2_SO_4_, NH_4_NO_3_, and NH_4_Cl were added to separate cultures at 30°C to observe the effects of the N source. The medium was cultured at 20°C, 25°C, 30°C, 35°C, or 40°C to observe the effects of temperature. Samples were taken at 3, 7, 12, 15, 18, and 21 days after inoculation to determine the concentration of QNC and to calculate the degradation rate using the following equation: QNC degradation rate (%) = (1 − experimental measurement value/control measurement value) × 100. Each treatment was performed in triplicate. The control was the treatment without inoculation.

### (iii) Degradation of QNC by strain D under optimal culture conditions.

Liquid MSM was prepared with a QNC concentration of 50 mg · liter^−1^, and the pH was adjusted to 6. The N source, (NH_4_)_2_SO_4_, was added with 0.1% yeast extract powder and 7% bacterial suspension. The medium was cultured at 30°C and 180 rpm. Samples were taken at 3, 7, 12, 15, 18, and 21 days after inoculation to determine the QNC concentration and to calculate the degradation rate. The OD_600_ value of the bacterial suspension was determined using an UV spectrophotometer (UV2600; Shimadzu, Japan). Each treatment was performed in triplicate. The control was the treatment without bacteria.

### Determination of degradation products.

Strain D was cultured under optimal conditions for 21 days, and samples were taken at 3, 7, 12, 15, 18, and 21 days after inoculation. The QuEChERS method was used to extract QNC and its degradation products. The degradation products of QNC were detected by HPLC–Q-TOF MS (Agilent, USA). The HPLC parameters were set as follows: XSelect HSS T3 column (2.1 mm by 150 mm by 3.5 μm; American Waters); column temperature, 45°C; mobile phase, aqueous solution containing 0.1% formic acid (vol/vol) (solvent A) and acetonitrile (solvent B) for HPLC; gradient elution, 0 to 5 min, 10% to 40% solvent B; 5 to 11 min, 40% to 95% solvent B; 11 to 15 min, 95% solvent B; 15 to 15.1 min, 95% to 10% solvent B; 15.1 to 21 min, 10% solvent B; flow rate, 300 μl · min^−1^; injection volume, 5 μl. MS parameter settings were as follows: electrospray ionization; positive- and negative-ion full-scan modes; mass scan range, *m/z* 50 to 1,200; mass scan rate, 4 spectra · s^−1^. The parameters set for positive- and negative-ion scanning were as follows: drying gas temperature, 350°C; drying gas flow rate, 8 liter · min^−1^; sheath gas temperature, 280°C; sheath gas flow rate, 11 liter · min^−1^; atomizing gas pressure, 40 psi; cone fragmentation voltage, 130 V; skimmer voltage, 40 V; radiofrequency voltage, 750 V; positive-ion mode: capillary voltage, 4,000 V; nozzle voltage, 500 V; negative-ion mode: capillary voltage, 3,500 V; nozzle voltage, 1,000 V.

### Effects of strain D on QNC degradation and rice plant growth in a simulated QNC-contaminated rice paddy. (i) Pretreatment for the pot experiment.

Paddy soil was dried and sieved (2 mm), and a portion of the soil was sterilized. Strain D was inoculated into liquid LB medium, cultured at 30°C and 180 rpm for 48 h, washed twice in physiological saline solution, and resuspended in sterilized water to yield a bacterial suspension for later use. For pesticide preparation, two quantities of QNC mother liquor, one with an active ingredient concentration of 375 g · hm^−2^ · a^−1^ (the manufacturer-recommended dose) and one with an effective ingredient concentration of 750 g · hm^−2^ · a^−1^ (2 times the manufacturer-recommended dose), were prepared for use. Rice seeds were sterilized and soaked to accelerate germination and were cultivated to the two-leaf–one-heart stage in an artificial incubator. Rice seedlings of uniform growth were selected for transplanting.

### (ii) Pot experiment.

Selected rice seedlings of a variety that grows consistently and is robust were transplanted into potting buckets (25 cm by 20 cm) with 6 kg of soil in each bucket. After 7 days, 8 treatments were administered, as follows. For treatment nCK-1, normal soil was sprayed with the manufacturer-recommended dose of QNC (375 g · hm^−2^ · a^−1^). For treatment nT-1, normal soil was sprayed with the manufacturer-recommended dose of QNC to which the bacterial suspension had been added and evenly mixed to a concentration of 3.38 × 10^8^ CFU · g^−1^. For treatment nCK-2, normal soil was sprayed with 2 times the manufacturer-recommended dose of QNC (750 g · hm^−2^ · a^−1^). For treatment nT-2, normal soil was sprayed with 2 times the manufacturer-recommended dose of QNC to which the bacterial suspension had been added and evenly mixed to a concentration of 3.38 × 10^8^ CFU · g^−1^. For treatment sCK-1, sterilized soil was sprayed with the manufacturer-recommended dose of QNC. For treatment sT-1, sterilized soil was sprayed with the manufacturer-recommended dose of QNC to which the bacterial suspension had been added and evenly mixed to a concentration of 3.38 × 10^8^ CFU · g^−1^. For treatment sCK-2, sterilized soil was sprayed with 2 times the manufacturer-recommended dose of QNC. For treatment sT-2, sterilized soil was sprayed with 2 times the manufacturer-recommended dose to which the bacterial suspension had been added and mixed evenly to a concentration of 3.38 × 10^8^ CFU · g^−1^. Each treatment was performed three times. After treatment, random multipoint sampling was used at 0, 3, 7, 14, 21, and 28 days to collect rice plants and soil samples. Soil samples were analyzed to detect QNC residues. The plant height, root length, stem dry weight, stem fresh weight, root dry weight, and root fresh weight of rice plant samples collected at 7 days and 21 days were measured. Then SPSS 25 software was used to perform one-way analysis of variance and multiple-comparison tests.

### Data availability.

The complete 16S rRNA gene sequence of *Cellulosimicrobium* sp. strain D was deposited in GenBank under the accession no. MW404399.
